# Accelerating drug discovery at Exelixis through cryo-EM

**DOI:** 10.1063/4.0001068

**Published:** 2025-10-27

**Authors:** William J Nicolas

**Affiliations:** Exelixis, Alameda, CA, USA

## Abstract

Structure based drug design is now an established paradigm for the industrious task of making and optimizing novel therapeutics. The cryo- electron microscopy (cryo-EM) “resolution revolution”, and the recent automation improvements in data acquisition and processing make cryo-EM single particle analysis a pivotal method for high resolution structural enablement at an industrial scale. At Exelixis, our brand new cryo-EM facility allows a combination of efficient structural enablement within days and high flexibility. This allows i) efficient and reliable continuous support when improving the interaction between a compound and a target, and ii) quick on-boarding and optimization of new targets.

